# Metabolomics Profiling to Investigate the Pharmacologic Mechanisms of Berberine for the Treatment of High-Fat Diet-Induced Nonalcoholic Steatohepatitis

**DOI:** 10.1155/2015/897914

**Published:** 2015-04-22

**Authors:** Jian Li, Zezhou Liu, Mingxing Guo, Kejia Xu, Miao Jiang, Aiping Lu, Xiaoyan Gao

**Affiliations:** ^1^School of Basic Medical Sciences, Beijing University of Chinese Medicine, Beijing 100029, China; ^2^Center of Scientific Experiment, Beijing University of Chinese Medicine, Beijing 100029, China; ^3^Institute of Basic Research of Clinical Medicine, China Academy of Chinese Medical Sciences, Beijing 100700, China; ^4^School of Chinese Medicine, Hong Kong Baptist University, Kowloon, Hong Kong

## Abstract

*Objective*. Berberine has been used to treat nonalcoholic steatohepatitis (NASH), which has been addressed in many studies. In this study, we investigated the molecular pharmacology mechanisms of berberine using metabolomic techniques. *Methods*. Sprague-Dawley rats were randomly divided into three groups (10 rats in each group): (i) normal control group; (ii) high-fat diet- (HFD-) induced NASH model group; and (iii) HFD berberine-treated group (i.d. 200 mg/kg). The handling procedure lasted eight weeks. Then, UPLC-Q-TOF/MS techniques coupled with histopathology and biochemical analyses were adopted to explore the mechanisms of berberine on the protective effects against NASH. *Key Findings*. (i) According to conventional test results, berberine treatment plays a fighting role in HFD-induced NASH due to its beneficial effects against insulin resistance, inflammation, and lipid metabolism. (ii) Based on UPLC-Q-TOF/MS techniques, metabolic profiles that involved sphingomyelin (SM), phosphatidylcholine (PC), lysophosphatidylcholine (LysoPC), 13-hydroperoxy-9, 11-octadecadienoic acid (13-HpODE), eicosatrienoic acid, docosatrienoic acid, and eicosenoic acid could provide potential metabolic biomarkers to address the pharmacological mechanisms of berberine. *Conclusions*. The parts of molecular pharmacological mechanisms of berberine for NASH treatment are related to the regulation of metabolic disruption involving phospholipid and unsaturated fatty acids in rats with NASH.

## 1. Introduction

Nonalcoholic fatty liver disease (NAFLD) is an increasingly recognized disease state in which lipids (triglycerides) accumulate in the hepatocytes in the absence of excessive alcohol consumption [1, 2]. The earliest stage of NAFLD is hepatic steatosis, which is often contained. However, it can progress to nonalcoholic steatohepatitis (NASH) if inadequate treatment or poor prognosis occurs. The operational definitions for NASH have remained uncertain until now. Thus, NASH is distinguished from simple steatosis by the presence of hepatocyte ballooning, cell death, inflammatory infiltrate, and/or collagen deposition in liver tissue [3]. As the worldwide trend currently continues towards an increased prevalence of NAFLD [4–7], the pathogenesis, diagnosis, and specific therapeutic strategies have duly gained the critical attention of researchers.

NASH is closely related to metabolic syndromes linked to obesity, insulin resistance, metabolic disorders, free radical reactions, and dyslipidemia, among others [8, 9]. Different kinds of pharmacologic agents, such as insulin sensitizers, antioxidants,* n-3 *polyunsaturated fatty acids, ursodeoxycholic acid, lipase inhibitors, and lipid-lowering drugs, are therefore thought to have beneficial effects on the treatment of NASH [10]. However, due to undesirable side effects and the limited effectiveness of current chemical drugs for NAFLD, researchers have focused on the development of natural drugs from herbs.

Berberine (C_20_H_18_NO_4_), an isoquinoline alkaloid, is one of the main bioactive constituents of* Rhizoma coptidis*, which has been used to treat diabetes in China for over a thousand years [11]. At present, many derivatives of berberine, for example, berberine hydrochloride, berberine sulfate, and berberine citrate, have been developed for a patented drug used as an antimicrobial drug for treating gastroenteritis and bacillary dysentery in clinics [12]. Moreover, some novel pharmacological properties were discovered, which mainly concern metabolic diseases, such as obesity [13–15], type 2 diabetes [16–18], and NAFLD [19–21]. Although the pharmacological mechanisms of berberine have been inferred from many studies, the data were mostly obtained from routine examinations, such as mediating insulin resistance, regulating the adenosine monophosphate-activated protein kinase (AMPK) pathway, and modifying the gut microenvironment [19]. The detailed metabolic mechanisms of berberinefor treating NASH have not been reported to date.

Compared with other conventional methods, is a better approach for studying the pathogenesis of diseases and pharmacological mechanisms by monitoring many endogenous “low-molecular-weight” metabolites using data acquiring methods, such as LC/MS, NMR, and GC [22, 23]. Here, we report a metabolomics study on a high-fat diet- (HFD-) induced NASH animal model using UPLC-Q-TOF/MS techniques coupled with histopathology and biochemical analysis to reveal the mechanisms of berberine on the protective effects against NASH.

## 2. Materials and Methods

### 2.1. Chemicals and Reagents

Standards and HFD were purchased from Charles River Company. The feed formula of HFD consists of 88% basic feed (58% fat, 12.4% protein, 17% carbohydrate, and 0.6% vitamin and mineral), 10% lard, and 2% cholesterol. Berberine was ordered from Nanjing Ze-Lang Pharmaceutical Technology Co., Ltd. (Nanjing, China). Colorimetric kits of triglyceride HPLC grade formic acid were obtained from Sigma Chemical Co., Ltd. (St. Louis, MO, USA). Methanol and acetonitrile (HPLC grade) were acquired from Fisher Corporation (Michigan, USA). Ultrahigh purity water was prepared by Millipore-Q SAS 67120MOLS HEIM (France).

### 2.2. Animal Treatment and Sampling

A total of 30 male Sprague-Dawley rats (150 ± 10 g) were commercially obtained from the Charles River Company (Beijing, China, Rodent license number SYXK 11-00-0039). Rats were randomly divided into three groups: (i) a normal control group with a standard diet and vehicle* per os* by gavage (NC), (ii) an HFD group with a vehicle (M), and (iii) an HFD group with berberine-treated group receiving HFD coupled intragastric administration with berberine (Ber, i.d. 200 mg/kg body weight). The handing procedure lasted eight weeks. Blood samples were collected using an abdominal aorta puncture and obtaining serum. Parts of liver tissue were excised and formalin-fixed immediately, and some liver tissue was frozen by liquid nitrogen.

### 2.3. Biochemical Assays

Fasting serum alanine aminotransferase (ALT), aspartate aminotransferase (AST), triglyceride (TG), total cholesterol (TC), blood glucose, and low density lipoprotein cholesterol (LDL-C) were measured by automated procedures according to the manufacturers' protocols (BioSino Bio-tech and Science Inc.). TNF-*α* and IL-6 concentrations were analyzed using commercially available ELISA kits (Shanghai Yanji Bio-tech Co., Ltd.). Fasting insulin concentrations were measured by a rat insulin radioimmunoassay kit, and whole body insulin sensitivity was estimated using the homeostasis model assessment of insulin resistance (HOMA-IR) using the following formula: HOMA-IR = fasting glucose (mmol/L) × fasting insulin (*μ*U/mL)/22.5.

### 2.4. Histopathological Evaluation

Liver tissue paraffin sections (4 *μ*m) were stained with hematoxylin and eosin (H&E). The frozen section was dyed using red O oil. The pathological diagnosis standard of NASH depends on the NAFLD activity score (NAS). The diagnosis of NASH is clear with a NAS ≥ 5 and is excluded when NAS < 3, and values in between have the possibility of NASH [24].

### 2.5. Metabolomic Analysis

The global, unbiased metabolic profiling platform was based on a Waters ACQUITY Ultra Performance Liquid Chromatography (UPLC) system coupled to a Xevo G2-Q-TOF/MS. This platform was described in detail in our previous publication [25]. In brief, 200 *μ*L of each serum sample was thawed on ice and extracted by methanol protein precipitation. The supernatant (400 *μ*L) was transferred to a clean tube and evaporated until dry under a gentle stream of nitrogen. The residue was reconstituted with 100 *μ*L of ultrahigh purity water and transferred to an autosampler vial. UPLC-Q-TOF/MS was carried out using an ACQUITY UPLC HSS T3 column (2.1 × 100 mm, 1.8 *μ*m, UK) maintained at 45°C. The gradient program commenced with 100% of a 0.1% formic acid in water for 1 min at a flow rate of 0.30 mL/min which then changed to 60% methanol linearly within 8 min and changed to 100% methanol linearly within 2 min. This was then held for 2 min before finally reverting back to 100% of 0.1% formic acid in water. Once these initial settings were reached, the column was reequilibrated for 2 min. The injection volume was 1 *μ*L. The ESI ionization source parameters used were as follows: capillary voltage, 3000 V for positive ion mode and 2500 V for negative ion mode; cone voltage, 30 V for positive ion mode and 25 V for negative ion mode; collision energy, 6 eV; desolvation gas, 750 L/h; cone gas, 50 L/h; desolvation temperature, 350°C; and source temperature, 100°C. Full scan mode was employed in the mass range of 50–1200 amu. Leucine-enkephalin was used as the lock mass. The collision energy was set at 20 eV. The UPLC/MS raw data were preprocessed using the Micromass MarkerLynx Applications Manager version 4.0 (Waters Corp., Milford, USA). The area of each peak, after being recognized and aligned, was normalized to the summed total ion intensity of each chromatogram.

The resulting data were then exported into EZinfo 2.0 software (Waters Corporation, Milford, MA, USA) for PCA and OPLS-DA. Student's* t*-test and random forest analysis were performed with MetaboAnalyst 2.0 (http://www.metaboanalyst.ca/) to test the ability of the metabolomic data in correctly classifying the samples into their respective groups. The MS^E^ technique was used to assign the metabolite peaks. Some available biochemical databases such as HMDB (http://www.hmdb.ca/), KEGG (http://www.genome.jp/kegg/), METLIN (http://metlin.scripps.edu/), LIPID MAPS (http://www.lipidmaps.org/), and ChemSpider (http://www.chemspider.com/) were used to analyze and explain potential biomarkers. To assess the ability to classify subjects as equivalents to the normal control with NASH model and with berberine treatment, a random forest analysis was performed using the complete metabolomic data.

### 2.6. Statistical Analysis

The statistical analysis of the relative intensity of biomarkers was performed by SPSS 17.0. The integration areas of the detected metabolites with high VIP values were first tested for the normality of the distribution. If the distribution followed the normality assumption, parametric Student's* t*-test was applied; otherwise, a nonparametric Mann-Whitney *U* test was performed to detect statistically significant metabolites that were increased or decreased between groups. Differences were considered significant at a value of *P* < 0.05. Statistical analysis of one-way ANOVA was also performed on the biochemical analysis data.

## 3. Results and Discussion

### 3.1. Effects of Berberine on Liver Steatosis, Inflammation, and Serum Parameters

**Figure 1:**
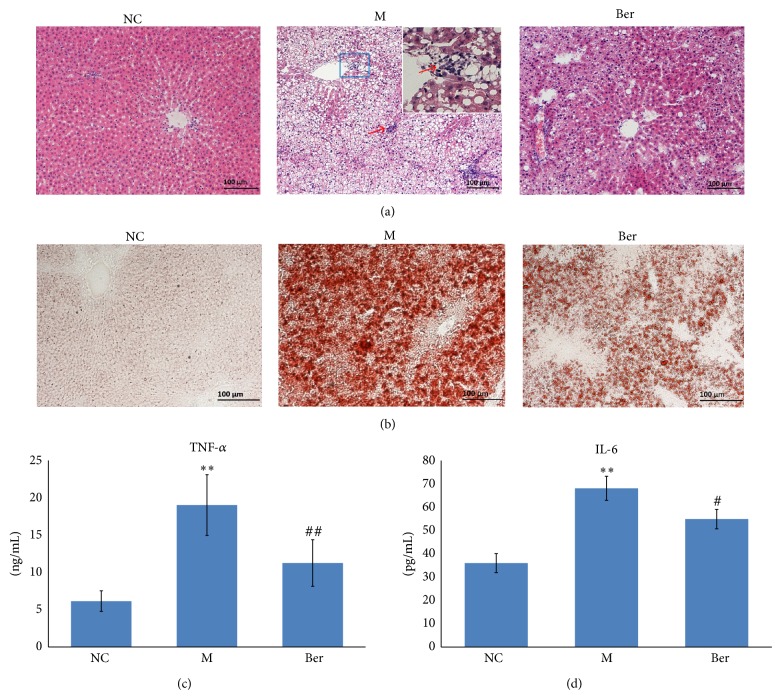
Effects of berberine on hepatic pathological, and the level of serum proinflammatory (TNF-*α*, IL-6). (a) Liver tissue paraffin section and H&E staining; (b) frozen section and red O oil staining; (c) serum TNF-*α* and IL-6 level detected by ELISA.

Compared with the normal control, administration of HFD to rats caused a significant increase in body weight. However, we observed a marked reduction in body weight gain following berberine treatment. Moreover, berberine was well tolerated in all rats without any adverse effects (data not shown). To explore whether berberine exerted beneficial effects on liver histopathology, paraffin-embedded specimens and frozen tissues were analyzed by H&E and red O oil staining. The results showed that HFD caused a marked accumulation of fat in hepatocytes (red O oil staining demonstrated steatosis affected most of the hepatocytes, Figure 1(b)) and an evident infiltration of inflammatory cells in foci or in surrounding groups of hepatocytes as evidenced by arrows (Figure 1(a)). Treatment with berberine resulted in a general improvement of steatosis and inflammation associated with the HFD (Figures 1(a) and 1(b)). No alterations were shown in the livers of rats fed with the standard diet. The results of histopathological scores indicated that berberine treatment plays a fighting role in HFD-induced NASH. In HFD-fed rats, TNF-*α*, the cytokines involved in the development of inflammatory responses, was significantly higher in serum but lower in the concentration in the treatment of berberine (*P* < 0.01, Figure 1(c)). Similarly, IL-6, another inflammation-related cytokine, showed higher levels in HFD rats and was downregulated by berberine treatment (*P* < 0.05, Figure 1(d)). Biochemical serum parameters are reported in Table 1. The results showed that serum levels of ALT, AST, CHO, TG, and LDL-C were significantly higher in HFD-fed rats (*P* < 0.05 or *P* < 0.01). However, all these parameters (except LDL-C) were lower in berberine-treated rats (*P* < 0.05 or *P* < 0.01). Compared to the normal control, HFD-fed rats showed a remarkable increase in fasting glucose (*P* < 0.05). Interestingly, the glucose alteration could be affected by the treatment of berberine without changes in fasting insulin levels. The homeostasis model assessment for insulin resistance (HOMA-IR) was lower in the berberine-treated group compared with that of HFD group (*P* < 0.05). Our results are in agreement with most studies in which berberine has a positive therapeutic effect on NAFLD due to its beneficial effects against insulin resistance, inflammation, and lipid metabolism [19, 26–28].

### 3.2. Metabolomic Analysis

**Figure 2:**
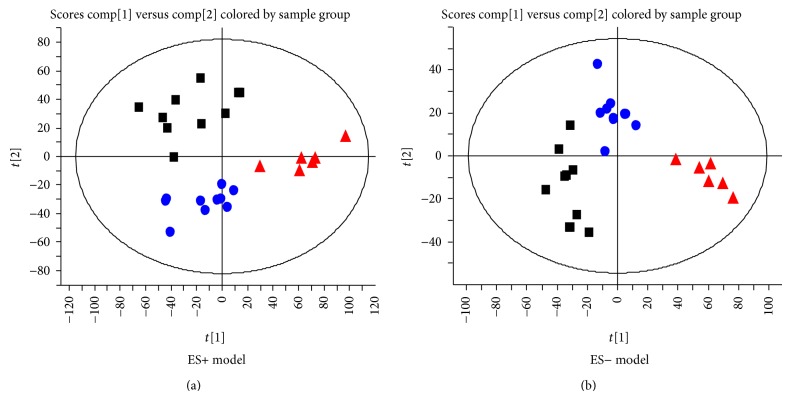
PCA scores plots discriminating HFD-fed rats from normal control rats and berberine treatment rats: (a) positive ion mode; (b) negative ion mode.* Note.* ■ showed normal control rats; ▲ showed HFD-induced NASH rats; ● showed NASH combined with berberine treatment rats.

**Figure 3:**
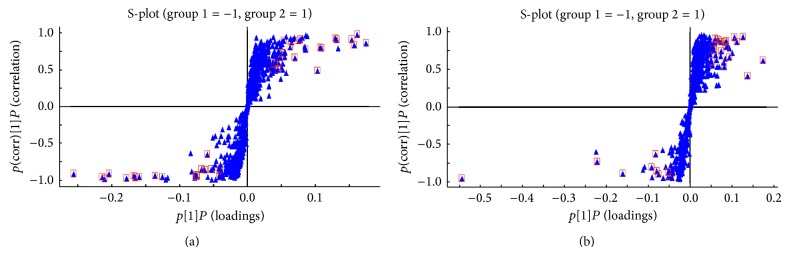
The results of multiple pattern recognition of serum biomarkers between normal control rats and HFD-induced NASH rats: (a) OPLS-DA score plot under positive ion mode; (b) OPLS-DA score plot under negative ion mode.

**Figure 4:**
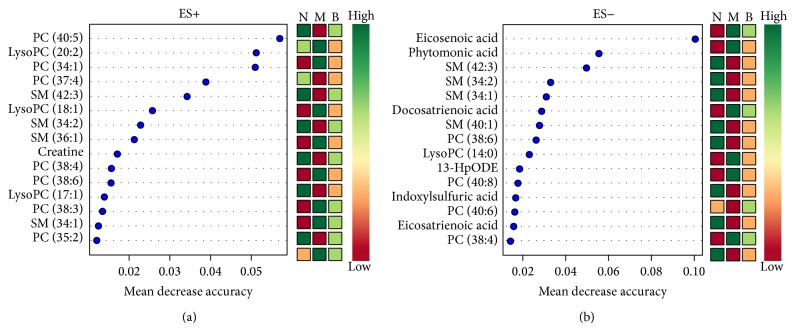
Random forest importance plot for each subjects: (a) positive ion mode; (b) negative ion mode.* Note.* N: normal control rat; M: NASH rat; B: berberine treatment rat.

**Figure 5:**
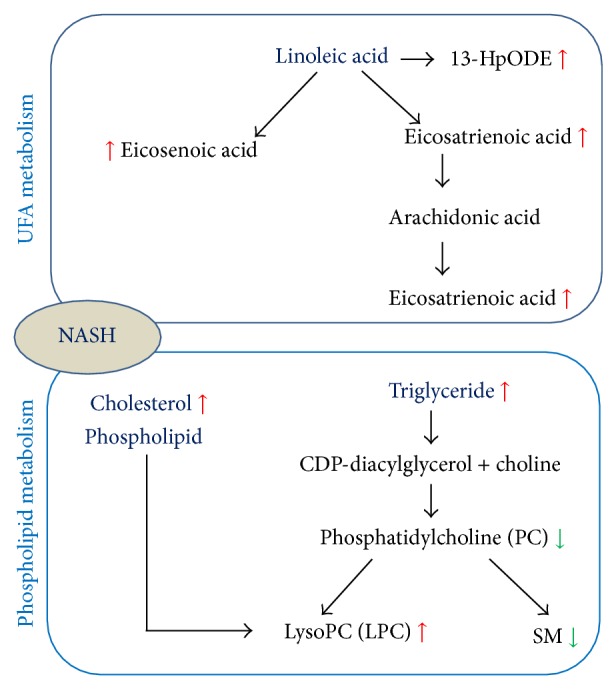
The metabolic network profile. The map was gained by analyzing known metabolic pathways.

A total of 1494 ions peaks were obtained from UPLC-Q-TOF/MS spectra (870 in positive mode and 624 in negative mode, data not shown). To gain an overview of the serum metabolic profile, PCA and OPLS-DA were used in the subsequent data analysis. The score plots of PCA showed well-delineated clusters and separation trends of the normal control group, the HFD group, and HFD combined with berberine-treated group in both positive ion mode and negative ion mode, highlighting the disease diagnostic potential and drug intervention effect (Figure 2). When loading the plot, we considered and selected key metabolites that predominantly accounted for variability along two vectors in the HFD-induced NASH model group relative to the normal control group based on the VIP values (VIP > 1) by two component OPLS-DA models (Figure 3). The univariate statistical method, Student's* t*-test, was performed on all serum features derived from calculations between the HFD-induced NASH group and normal control group, and berberine-treated group and model group, respectively. The variables selected were those with statistical significance (*P* < 0.05). A total of 72 metabolites (45 in positive mode and 27 in negative mode) were identified and listed (see supplementary data Table S1 in Supplementary Material available online at http://dx.doi.org/10.1155/2015/897914). To address the perturbation degrees, we further used “random forest” analysis to filter significantly distinguished metabolites among three groups.  Figure 4 showed the important features ranked by random forest. The features were ranked by the mean decrease in classification accuracy when they are permuted. In the context of them, we selected the potential serum metabolic biomarkers and listed them in Table 2. In brief, the levels of sphingomyelin (SM 34:1; 34:2; 36:1; 42:3) and phosphatidylcholine (PC 34:1; 37:4; 38:3; 38:4; 38:6; 40:5; 40:6; 40:8) were significantly depressed (*P* < 0.01; *P* < 0.05), while the levels of lysophosphatidylcholine (LPC 14:0; 17:1; 18:1; 20:2), 13-hydroperoxy-9,11-octadecadienoic acid (13-HpODE), eicosatrienoic acid, phytomonic acid, docosatrienoic acid, and eicosenoic acid were significantly higher in HFD rats than those of the normal control rats (*P* < 0.01; *P* < 0.05). On the contrary, berberine could cause a systemic recovery from the HFD-induced metabolic perturbation in rats. In the context of changed metabolic profiles, we sketched the perturbed metabolic network associated with NASH (Figure 5). Figure 5 shows that the metabolic pathways of phospholipid and unsaturated fatty acid (UFA) correlated metabolites were involved in HFD-induced metabolic disorders.

According to the traditional view, 3-indoxyl-sulfuric acid, a renal toxin that accumulates in blood, is derived from tryptophan degradation via indole and indoxyl and conjugated with sulfate. PC is an essential phospholipid in mammalian cells and tissue that is synthesized via the choline or phosphatidylethanolamine pathway. The liver is principally involved in the metabolism and release of PC into circulation. Usually, plasma PC is rapidly metabolized by phospholipase A2 to release the fatty acid and LPC into the plasma pool for distribution to extrahepatic tissues. LPC is an important signaling molecule with diverse biological functions. Reports by other investigators have shown that NAFLD was associated with downregulated PC and upregulated LPC level, which supported that PC and LPC might be effectors involved in mediating cellular inflammatory response and insulin resistance [29, 30]. SM is a type of sphingophospholipid found in animal cell membranes and is derived from PC and hydrolyzed by sphingomyelinases [31, 32]. It has been shown that dietary supplementation with SM could decrease total hepatic cholesterol and triglyceride level and reduce intestinal cholesterol absorption, helping to maintain lipid homeostasis [33]. Our data agree with the mentioned report, and the results suggested that berberine could be used against HFD by inducing NASH via directly perturbing the abnormal phospholipid metabolic pathway involving the relative content of PC, LPC, and SM.

In this experiment, multiple increased metabolites of UFA (13-HpODE, eicosatrienoic acid, phytomonic acid, docosatrienoic acid, and eicosenoic acid) were involved in the pathogenesis of HFD-induced NASH rats. As known, the mentioned UFAs are derived from linoleic acid (LA) [34, 35]. Except for the dietary resource, the increased serum level of the abovementioned metabolites of UFAs suggested upregulated endogenous LA, which is paradoxically associated with insulin sensitivity; some researchers showed that the increased proportion of LA in serum could improve insulin sensitivity [36–39] while others held the opposite view [40]. Although further studies might be required to clarify the role of UFA in NASH, altering the metabolism of UFA might provide an interesting system to study pharmacological mechanisms of berberine.

## 4. Conclusion

In this study, we investigated the effect of berberine on NASH in an HFD-fed rat model using in vivo UPLC-MS techniques. Our HFD model showed serum hypertriglyceridemia and hypercholesterolemia, confirming insulin resistant conditions. Moreover, the histopathological results from HFD-fed rats showed excessive accumulation of triglycerides with the lipid vacuoles occupying the hepatocytes with enhanced inflammatory responses. We demonstrated that berberine as a supplement could alleviate hepatic steatosis and pathological grade, reduce serum cholesterolemia and triglyceride, and prevent the development of insulin resistance in HFD-fed rats. In our strategy, as proposed in this study, global metabolic profiling was detected, suggesting a metabolic disruption associated with phospholipid and unsaturated fatty acids in the HFD-induced NASH rats. In contrast, berberine has led to a network switch in the metabolic profiles involving SM, PC, LPC, 13-HpODE, eicosatrienoic acid, docosatrienoic acid, and eicosenoic acid. These markers matched with the metabolic pathways of phospholipid and unsaturated fatty acid (UFA) correlated metabolites, which partly revealed the pharmacological mechanisms on NASH treatment using berberine.

Through our study in this paper alone, we cannot yet identify potential metabolites. As widely known, metabolite identification is a complex process in metabolomics and does not provide 100% coverage [41]. Currently, there are still many chromatographic peaks that cannot be identified in metabolomic data sets among the chromatography MS acquired data. The reasons are that (1) the metabolites have not been completely and experimentally characterized yet and the libraries and databases of experimental data applied for identification are not yet completed to reflect all known metabolites and that (2) not all metabolites known to be present can be purchased to construct mass spectral libraries to aid in the identification processes. Therefore, different levels of identification are available, and the reporting procedures for metabolite identification in metabolomics have been described by the metabolomics standards initiative. In this study, the molecular formula was identified by searching the public databases, such as HMDB (http://www.hmdb.ca/), KEGG (http://www.genome.jp/kegg/), ChemSpider (http://www.chemspider.com/), and METLIN (http://masspec.scripps.edu/). Therefore, identification of all detected metabolites is not currently achievable and is a significant limitation in metabolic profiling. Because obtaining standards for precise identification and quantification is a complex and time-consuming process, this part of the experiment will be our focus in a future study.

## Supplementary Material

Supplementary Table: Drug-induced variable metabolites in the serum of berberine treatment rats.

## Figures and Tables

**Table 1:** Change in serum parameters of rats fed on a standard diet (NC), high-fat diet (M), or HFD with the berberine (Ber) treatment for eight weeks ($\overset{-}{x}\pm s$, *n* = 10).

Index	NC	M	Ber
ALT (U/L)	19.49 ± 4.68	40.67 ± 9.47^**^	20.92 ± 5.56^##^
AST (U/L)	199.88 ± 30.74	390.60 ± 59.13^**^	281.57 ± 70.86^##^
CHO (mmol/L)	1.68 ± 0.19	2.81 ± 0.79^*^	2.07 ± 0.22^#^
TG (mmol/L)	0.56 ± 0.04	1.65 ± 0.12^*^	0.65 ± 0.13^##^
LDL-C (mmol/L)	0.16 ± 0.02	0.81 ± 0.42^*^	0.54 ± 0.38
Glucose (mmol/L)	3.51 ± 0.67	4.74 ± 2.20^*^	3.24 ± 1.17^#^
Insulin (mIU/L)	16.67 ± 4.0	17.09 ± 2.40	15.77 ± 3.53
HOMA index	2.6 ± 0.68	3.6 ± 3.02^*^	2.3 ± 0.75^#^

Note: ^∗^ *P* < 0.05, compared with NC group; ^∗∗^ *P* < 0.01, compared with NC group; ^#^ *P* < 0.05 and ^##^ *P* < 0.01, compared with M group.

**Table 2:** Altered metabolites in serum samples of the nonalcoholic steatohepatitis model (M) and berberine (Ber) treatment group (PC: phosphatidylcholine; LysoPC: lysophosphatidylcholine; SM: sphingomyelin; 13-HpODE: 13-hydroperoxy-octadecadienoic acid).

tR	*m*/*z*	VIP	ESI model	Formula	Metabolites	Fold changes		Classify
						M/NC	Ber/M	
8.45	468.31/512.29	2.17/1.34	[M+H]^+^/[M+FA-H]^−^	C_22_H_46_NO_7_P	LysoPC (14:0)	**↑**	**↓**	Glycerophospholipids
8.46	311.22	1.09	[M-H]^−^	C_18_H_32_O_4_	13-HpODE	**↑**	**↓**	Linoleic acids
8.73	508.34	1.22	[M+H]^+^	C_25_H_50_NO_7_P	LysoPC (17:1)	**↑**	**↓**	Glycerophospholipids
8.88	522.36	4.93	[M+H]^+^	C_26_H_52_NO_7_P	LysoPC (18:1)	**↑**	**↓**	Glycerophospholipids
8.96	548.37	1.84	[M+H]^+^	C_28_H_54_NO_7_P	LysoPC (20:2)	**↑**	**↓**	Glycerophospholipids
9.25	305.25	4.05	[M-H]^−^	C_20_H_34_O_2_	Eicosatrienoic acid	**↑**	**↓**	Fatty acids
9.50	295.26	1.19	[M-H]^−^	C_19_H_36_O_2_	Phytomonic acid	**↑**	**↓**	Fatty acids
9.56	333.28	1.06	[M-H]^−^	C_22_H_38_O_2_	Docosatrienoic acid	**↑**	**↓**	Fatty acids
9.68	309.28	2.28	[M-H]^−^	C_20_H_38_O_2_	Eicosenoic Acid	**↑**	**↓**	Fatty acids
10.03	745.55/701.55	1.48/1.93	[M+FA-H]^−^/[M+H]^+^	C_39_H_77_N_2_O_6_P	SM (34:2)	**↓**	**↑**	Sphingolipids
10.30	830.57/874.56	1.17/1.04	[M+H]^+^/[M+FA-H]^−^	C_48_H_80_NO_8_P	PC (40:8)	**↓**	**↑**	Glycerophospholipids
10.37	747.57	3.08	[M+FA-H]^−^	C_39_H_79_N_2_O_6_P	SM (34:1)	**↓**	**↑**	Sphingolipids
10.38	703.58	4.74	[M+H]^+^	C_39_H_79_N_2_O_6_P	SM (34:1)	**↓**	**↑**	Sphingolipids
10.52	806.57	5.08	[M+H]^+^	C_46_H_80_NO_8_P	PC (38:6)	**↓**	**↑**	Glycerophospholipids
10.53	850.56	2.55	[M+FA-H]^−^	C_46_H_80_NO_8_P	PC (38:6)	**↓**	**↑**	Glycerophospholipids
10.92	796.58	1.87	[M+H]^+^	C_45_H_82_NO_8_P	PC (37:4)	**↓**	**↑**	Glycerophospholipids
10.94	731.61	1.56	[M+H]^+^	C_41_H_83_N_2_O_6_P	SM (36:1)	**↓**	**↑**	Sphingolipids
11.15	834.60/878.59	4.45/2.03	[M+H]^+^/[M+FA-H]^−^	C_48_H_84_NO_8_P	PC (40:6)	**↓**	**↑**	Glycerophospholipids
11.40	836.62	2.27	[M+H]^+^	C_48_H_86_NO_8_P	PC (40:5)	**↓**	**↑**	Glycerophospholipids
11.60	812.62	6.25	[M+H]^+^	C_46_H_86_NO_8_P	PC (38:3)	**↑**	**↓**	Glycerophospholipids
12.05	811.67/855.66	3.81/2.24	[M+H]^+^/[M+FA-H]^−^	C_47_H_91_N_2_O_6_P	SM (42:3)	**↓**	**↑**	Sphingolipids

*Note.* The data were calculated using the integrated peak areas. The value represents fold change. VIP was obtained from OPLS-DA with a threshold of 1.0. The up and down arrows indicate a respective increased or decreased concentration of each metabolite in the HFD group versus normal control (M/NC) and berberine-treated versus HFD group (Ber/M).

## References

[1] Angulo P. (2002). Medical progress: nonalcoholic fatty liver disease. *The New England Journal of Medicine*.

[2] Angulo P. (2005). Nonalcoholic fatty liver disease. *Revista de Gastroenterología de México*.

[3] Cohen J. C., Horton J. D., Hobbs H. H. (2011). Human fatty liver disease: old questions and new insights. *Science*.

[4] Wang Z., Xia B., Ma C., Hu Z., Chen X., Cao P. (2007). Prevalence and risk factors of fatty liver disease in the Shuiguohu district of Wuhan city, central China. *Postgraduate Medical Journal*.

[5] Fan J.-G., Zhu J., Li X.-J. (2005). Prevalence of and risk factors for fatty liver in a general population of Shanghai, China. *Journal of Hepatology*.

[6] Hamaguchi M., Kojima T., Takeda N. (2005). The metabolic syndrome as a predictor of nonalcoholic fatty liver disease. *Annals of Internal Medicine*.

[7] Browning J. D., Szczepaniak L. S., Dobbins R. (2004). Prevalence of hepatic steatosis in an urban population in the United States: impact of ethnicity. *Hepatology*.

[8] Tilg H., Moschen A. R. (2010). Evolution of inflammation in nonalcoholic fatty liver disease: the multiple parallel hits hypothesis. *Hepatology*.

[9] Pacifico L., Nobili V., Anania C., Verdecchia P., Chiesa C. (2011). Pediatric nonalcoholic fatty liver disease, metabolic syndrome and cardiovascular risk. *World Journal of Gastroenterology*.

[10] Schwenger K. J. P., Allard J. P. (2014). Clinical approaches to non-alcoholic fatty liver disease. *World Journal of Gastroenterology*.

[11] Li J.-C., Shen X.-F., Meng X.-L. (2013). A traditional Chinese medicine JiuHuangLian (*Rhizoma coptidis* steamed with rice wine) reduces oxidative stress injury in type 2 diabetic rats. *Food and Chemical Toxicology*.

[12] Chang C.-H., Huang W.-Y., Lai C.-H. (2011). Development of novel nanoparticles shelled with heparin for berberine delivery to treat *Helicobacter pylori*. *Acta Biomaterialia*.

[13] Zhang X., Zhao Y., Zhang M. (2012). Structural changes of gut microbiota during berberine-mediated prevention of obesity and insulin resistance in high-fat diet-fed rats. *PLoS ONE*.

[14] Hu Y., Davies G. E. (2010). Berberine inhibits adipogenesis in high-fat diet-induced obesity mice. *Fitoterapia*.

[15] Kim W. S., Lee Y. S., Cha S. H. (2009). Berberine improves lipid dysregulation in obesity by controlling central and peripheral AMPK activity. *American Journal of Physiology—Endocrinology and Metabolism*.

[16] Xie X., Meng X., Zhou X., Shu X., Kong H. (2011). Research on therapeutic effect and hemorrheology change of berberine in new diagnosed patients with type 2 diabetes combining nonalcoholic fatty liver disease. *Zhongguo Zhong Yao Za Zhi*.

[17] Gu Y., Zhang Y., Shi X. (2010). Effect of traditional Chinese medicine berberine on type 2 diabetes based on comprehensive metabonomics. *Talanta*.

[18] Zhang H., Wei J., Xue R. (2010). Berberine lowers blood glucose in type 2 diabetes mellitus patients through increasing insulin receptor expression. *Metabolism: Clinical and Experimental*.

[19] Liu Y., Zhang L., Song H., Ji G. (2013). Update on berberine in nonalcoholic Fatty liver disease. *Evidence-Based Complementary and Alternative Medicine*.

[20] Chang X., Yan H., Fei J. (2010). Berberine reduces methylation of the MTTP promoter and alleviates fatty liver induced by a high-fat diet in rats. *Journal of Lipid Research*.

[21] Abdelmalek M. F., Sanderson S. O., Angulo P. (2009). Betaine for nonalcoholic fatty liver disease: results of a randomized placebo-controlled trial. *Hepatology*.

[22] Bjerrum J. T., Nielsen O. H., Hao F. (2010). Metabonomics in ulcerative colitis: diagnostics, biomarker identification, and insight into the pathophysiology. *Journal of Proteome Research*.

[23] Lindon J. C., Holmes E., Bollard M. E., Stanley E. G., Nicholson J. K. (2004). Metabonomics technologies and their applications in physiological monitoring, drug safety assessment and disease diagnosis. *Biomarkers*.

[24] Kleiner D. E., Brunt E. M., van Natta M. (2005). Design and validation of a histological scoring system for nonalcoholic fatty liver disease. *Hepatology*.

[25] Gao X., Guo M., Peng L. (2013). UPLC Q-TOF/MS-based metabolic profiling of urine reveals the novel antipyretic mechanisms of qingkailing injection in a rat model of yeast-induced pyrexia. *Evidence-Based Complementary and Alternative Medicine*.

[26] Xing L.-J., Zhang L., Liu T., Hua Y.-Q., Zheng P.-Y., Ji G. (2011). Berberine reducing insulin resistance by up-regulating IRS-2 mRNA expression in nonalcoholic fatty liver disease (NAFLD) rat liver. *European Journal of Pharmacology*.

[27] Zhou J. Y., Zhou S. W., Zhang K. B. (2008). Chronic effects of berberine on blood, liver glucolipid metabolism and liver PPARs expression in diabetic hyperlipidemic rats. *Biological and Pharmaceutical Bulletin*.

[28] Lee Y. S., Kim W. S., Kim K. H. (2006). Berberine, a natural plant product, activates AMP-activated protein kinase with beneficial metabolic effects in diabetic and insulin-resistant states. *Diabetes*.

[29] Arendt B. M., Ma D. W., Simons B. (2013). Nonalcoholic fatty liver disease is associated with lower hepatic and erythrocyte ratios of phosphatidylcholine to phosphatidylethanolamine. *Applied Physiology, Nutrition, and Metabolism*.

[30] Han M. S., Lim Y.-M., Quan W. (2011). Lysophosphatidylcholine as an effector of fatty acid-induced insulin resistance. *Journal of Lipid Research*.

[31] Li Z., Agellon L. B., Vance D. E. (2007). A role for high density lipoproteins in hepatic phosphatidylcholine homeostasis. *Biochimica et Biophysica Acta: Molecular and Cell Biology of Lipids*.

[32] Testi R. (1996). Sphingomyelin breakdown and cell fate. *Trends in Biochemical Sciences*.

[33] Chung R. W. S., Kamili A., Tandy S. (2013). Dietary sphingomyelin lowers hepatic lipid levels and inhibits intestinal cholesterol absorption in high-fat-fed mice. *PLoS ONE*.

[34] Klein P. D. (1959). Linoleic acid and cholesterol metabolism in the rat. II. Effects of dietary cholesterol on plasma and liver ester composition. *Archives of Biochemistry and Biophysics*.

[35] Klein P. D. (1958). Linoleic acid and cholesterol metabolism in the rat. I. The effect of dietary fat and linoleic acid levels on the content and composition of cholesterol esters in liver and plasma. *Archives of Biochemistry and Biophysics*.

[36] Kahleova H., Matoulek M., Bratova M. (2013). Vegetarian diet-induced increase in linoleic acid in serum phospholipids is associated with improved insulin sensitivity in subjects with type 2 diabetes. *Nutrition & Diabetes*.

[37] Parra P., Palou A., Serra F. (2010). Moderate doses of conjugated linoleic acid reduce fat gain, maintain insulin sensitivity without impairing inflammatory adipose tissue status in mice fed a high-fat diet. *Nutrition and Metabolism*.

[38] Ahrén B., Mari A., Fyfe C. L. (2009). Effects of conjugated linoleic acid plus *n*-3 polyunsaturated fatty acids on insulin secretion and estimated insulin sensitivity in men. *European Journal of Clinical Nutrition*.

[39] Moloney F., Yeow T.-P., Mullen A., Nolan J. J., Roche H. M. (2004). Conjugated linoleic acid supplementation, insulin sensitivity, and lipoprotein metabolism in patients with type 2 diabetes mellitus. *The American Journal of Clinical Nutrition*.

[40] Kawashima Y., Musoh K., Kozuka H. (1990). Peroxisome proliferators enhance linoleic acid metabolism in rat liver. Increased biosynthesis of *ω*6 polyunsaturated fatty acids. *Journal of Biological Chemistry*.

[41] Dunn W. B., Broadhurst D., Begley P. (2011). Procedures for large-scale metabolic profiling of serum and plasma using gas chromatography and liquid chromatography coupled to mass spectrometry. *Nature Protocols*.

