# Supplementary material for: Metabolomics Profiling to Investigate the Pharmacologic Mechanisms of Berberine for the Treatment of High-Fat Diet-Induced Nonalcoholic Steatohepatitis
Source: Evid Based Complement Alternat Med. 2015 Apr 22;2015:897914. doi: 10.1155/2015/897914 (PMC4421035; doi:10.1155/2015/897914)
Supplement: Supplementary file 1 — Supplementary Table: Drug-induced variable metabolites in the serum of berberine treatment rats. [file 897914.f1.doc]

**Supplementary** Table 1. Metabolites selected by OPLS-DA with VIP > 1

**Ion mode:**  **ES+**

| **No.** | **t_R_-m/z** | **VIP** | **Formula** | **Name** | **Relative peak area** | | | **Classify** |
| --- | --- | --- | --- | --- | --- | --- | --- | --- |
|  |  |  |  |  | **NC** | **HFD** | **HFD+Ber** |  |
| **1** | 0.88-132.0780 | 3.18 | 3.18 | Creatine | 102.49 | 47.97 | 101.36 | Amino Acids |
| **2** | 3.46-166.0873 | 1.32 | 1.32 | Phenylalanine | 34.89 | 24.70 | 37.94 | Amino Acids |
| **3** | 1.55-204.1240 | 1.66 | 1.66 | Acetylcarnitine | 43.48 | 25.88 | 36.71 | Fatty Acid Esters |
| **4** | 8.13-426.3217 | 1.42 | 1.42 | Cholic acid | 16.05 | 1.63 | 4.18 | Bile Acids |
| **5** | 8.14-355.2636 | 1.16 | 1.16 | 3-Hydroxychola-7,22-dien-24-oic acid | 10.77 | 1.03 | 2.67 | Bile Acids |
| **6** | 8.49-285.2224 | 1.10 | 1.10 | 15-HETE | 26.29 | 16.75 | 32.92 | Eicosanoids |
| **7** | 8.45-468.3087 | 2.17 | 2.17 | LysoPC (14:0) | 11.69 | 33.98 | 13.62 | Lysophospholipid |
| **8** | 8.57-494.3245 | 3.94 | 3.94 | LysoPC (16:1) | 37.88 | 111.13 | 46.33 | Lysophospholipid |
| **9** | 8.68-544.3403 | 3.20 | 3.20 | LysoPC (20:4) | 181.25 | 124.68 | 167.67 | Lysophospholipid |
| **10** | 8.70-520.3402 | 6.16 | 6.16 | LysoPC (18:2) | 172.04 | 342.71 | 203.64 | Lysophospholipid |
| **11** | 8.73-508.3404 | 1.22 | 1.22 | LysoPC (17:1) | 1.86 | 8.64 | 4.042 | Lysophospholipid |
| **12** | 8.80-496.3402 | 4.56 | 4.56 | LysoPC (16:0) | 594.22 | 484.44 | 543.16 | Lysophospholipid |
| **13** | 8.88-522.3559 | 4.93 | 4.93 | LysoPC (18:1) | 251.41 | 364.78 | 341.21 | Lysophospholipid |
| **14** | 8.96-548.3715 | 1.84 | 1.84 | LysoPC (20:2) | 22.42 | 39.57 | 17.60 | Lysophospholipid |
| **15** | 9.06-1047.7348 | 1.96 | 1.96 | LysoPC (18:0) | 141.60 | 114.95 | 134.88 | Lysophospholipid |
| **16** | 10.15-754.5374 | 1.15 | 1.15 | PC (34:4) | 1.09 | 7.80 | 1.13 | phospholipid |
| **17** | 10.27-780.5521 | 1.71 | 1.71 | PC (36:5) | 1.99 | 21.71 | 6.87 | phospholipid |
| **18** | 10.29-756.5535 | 3.64 | 3.64 | PC (34:3) | 20.73 | 81.92 | 25.52 | phospholipid |
| **19** | 10.30-830.5686 | 1.17 | 1.17 | PC (40:8) | 45.23 | 34.72 | 36.15 | phospholipid |
| **20** | 10.42-782.5691 | 5.28 | 5.28 | PC (36:4) | 41.65 | 166.18 | 27.18 | phospholipid |
| **21** | 10.52-806.5697 | 5.08 | 5.08 | PC (38:6) | 219.05 | 85.78 | 163.85 | phospholipid |
| **22** | 10.81-784.5858 | 7.41 | 7.41 | PC (36:3) | 83.58 | 350.12 | 189.22 | phospholipid |
| **23** | 10.92-796.5849 | 1.87 | 1.87 | PC (37:4) | 31.27 | 12.51 | 20.71 | phospholipid |
| **24** | 10.99-734.5693 | 2.60 | 2.60 | PC (32:0) | 92.26 | 55.39 | 80.66 | phospholipid |
| **25** | 11.00-772.5840 | 2.23 | 2.23 | PC (35:2) | 20.05 | 43.23 | 22.53 | phospholipid |
| **26** | 11.11-760.5853 | 4.77 | 4.77 | PC (34:1) | 156.09 | 260.83 | 212.53 | phospholipid |
| **27** | 11.15-834.6007 | 4.45 | 4.45 | PC (40:6) | 162.87 | 66.44 | 116.81 | phospholipid |
| **28** | 11.35-786.6005 | 6.01 | 6.01 | PC (36:2) | 284.46 | 456.47 | 336.26 | phospholipid |
| **29** | 11.40-836.6164 | 2.27 | 2.27 | PC (40:5) | 33.94 | 8.86 | 21.44 | phospholipid |
| **30** | 11.60-812.6160 | 6.25 | 6.25 | PC (38:3) | 27.89 | 208.05 | 124.39 | phospholipid |
| **31** | 11.63-746.6053 | 1.23 | 1.23 | PC (34:0) | 23.24 | 13.03 | 20.72 | phospholipid |
| **32** | 11.89-838.6317 | 1.19 | 1.19 | PC (40:4) | 12.59 | 3.69 | 9.49 | phospholipid |
| **33** | 11.93-788.6165 | 4.71 | 4.71 | PC (36:1) | 81.75 | 182.88 | 133.02 | phospholipid |
| **34** | 11.94-810.5986 | 2.17 | 2.17 | PC (38:4) | 28.28 | 50.09 | 41.33 | phospholipid |
| **35** | 12.15-814.6312 | 1.99 | 1.99 | PC (38:2) | 0.05 | 20.19 | 1.93 | phospholipid |
| **36** | 11.54-658.5406 | 1.49 | 1.49 | DG (38:6) | 15.29 | 1.79 | 4.38 | Glycerolipids |
| **37** | 12.16-610.5405 | 1.55 | 1.55 | DG (34:2) | 5.99 | 18.30 | 4.63 | Glycerolipids |
| **38** | 12.37-601.5188 | 1.16 | 1.16 | DG (36:3) | 1.56 | 8.53 | 2.46 | Glycerolipids |
| **39** | 9.13-815.6992 | 1.35 | 1.35 | SM (42:1) | 23.76 | 8.41 | 12.69 | Sphingolipids |
| **40** | 10.04-701.5589 | 1.93 | 1.93 | SM (34:2) | 40.38 | 21.85 | 25.89 | Sphingolipids |
| **41** | 10.38-703.5750 | 4.74 | 4.74 | SM (34:1) | 183.60 | 83.16 | 151.31 | Sphingolipids |
| **42** | 10.94-731.6054 | 1.56 | 1.56 | SM (36:1) | 23.08 | 11.07 | 28.22 | Sphingolipids |
| **43** | 12.05-811.6683 | 3.81 | 3.81 | SM (42:3) | 99.91 | 32.08 | 59.31 | Sphingolipids |
| **44** | 12.75-787.6678 | 2.22 | 2.22 | SM (40:1) | 42.71 | 18.23 | 31.99 | Sphingolipids |
| **45** | 12.86-813.6848 | 3.85 | 3.85 | SM (42:2) | 164.73 | 93.21 | 151.56 | Sphingolipids |

**Ion mode:**  **ES-**

| **No.** | **t_R_-m/z** | **VIP** | **Formula** | **Name** | **Relative peak area** | | | **Classify** |
| --- | --- | --- | --- | --- | --- | --- | --- | --- |
|  |  |  |  |  | **NC** | **HFD** | **HFD+Ber** |  |
| **1** | 3.46-164.0708 | 1.73 | C_9_H_11_NO_2_ | Phenylalanine | 57.55 | 32.79 | 54.29 | Amino Acids |
| **2** | 2.60-180.0656 | 1.99 | C_9_H_11_NO_3_ | Tyrosine | 61.66 | 29.07 | 55.39 | Amino Acids |
| **3** | 3.93-212.0011 | 1.62 | C_8_H_7_NO_4_S | Indoxylsulfuric acid | 26.39 | 6.53 | 28.51 | Indoles |
| **4** | 8.13-407.2791 | 2.38 | C_24_H_40_O_5_ | Cholic acid | 69.65 | 8.42 | 21.30 | Bile Acids |
| **5** | 8.54-391.2845 | 1.33 | C_24_H_40_O_4_ | Ursodeoxycholic acid | 24.28 | 5.27 | 15.74 | Bile Acids |
| **6** | 8.46-311.2205 | 1.09 | C_18_H_32_O_4_ | 13-HpODE | 4.82 | 13.79 | 6.58 | Lineolic |
| **7** | 8.45-512.2989 | 1.34 | C_22_H_46_NO_7_P | LysoPC (14:0) | 10.55 | 23.86 | 11.55 | Lysophospholipid |
| **8** | 8.57-538.3142 | 1.93 | C_24_H_48_NO_7_P | LysoPC (16:1) | 27.69 | 54.95 | 30.68 | Lysophospholipid |
| **9** | 8.67-588.3300 | 1.86 | C_28_H_50_NO_7_P | LysoPC (20:4) | 81.64 | 50.32 | 81.08 | Lysophospholipid |
| **10** | 8.69-564.3299 | 2.00 | C_26_H_50_NO_7_P | LysoPC (18:2) | 55.03 | 98.99 | 80.15 | Lysophospholipid |
| **11** | 8.94-554.3459 | 1.28 | C_25_H_52_NO_7_P | LysoPC (17:0) | 43.88 | 27.59 | 35.12 | Lysophospholipid |
| **12** | 9.05-568.3618 | 2.02 | C_26_H_54_NO_7_P | LysoPC (18:0) | 96.52 | 48.76 | 87.20 | Lysophospholipid |
| **13** | 9.12-303.2323 | 3.69 | C_20_H_32_O_2_ | Arachidonic acid | 658.86 | 484.20 | 475.22 | Fatty Acids |
| **14** | 9.25-305.2477 | 4.05 | C_20_H_34_O_2_ | Eicosatrienoic acid | 65.68 | 189.33 | 118.55 | Fatty Acids |
| **15** | 9.43-307.2633 | 1.96 | C_20_H_36_O_2_ | Eicosadienoic acid | 33.01 | 63.72 | 40.88 | Fatty Acids |
| **16** | 9.50-295.2630 | 1.19 | C_19_H_36_O_2_ | Phytomonic Acid | 2.82 | 13.14 | 7.91 | Fatty Acids |
| **17** | 9.56-333.2788 | 1.06 | C_22_H_38_O_2_ | Docosatrienoic Acid | 3.74 | 11.80 | 10.89 | Fatty Acids |
| **18** | 9.68-309.2790 | 2.28 | C_20_H_38_O_2_ | Eicosenoic Acid | 39.36 | 82.74 | 58.19 | Fatty Acids |
| **19** | 10.30-874.5615 | 1.04 | C_48_H_80_NO_8_P | PC (40:8) | 12.66 | 4.01 | 5.03 | phospholipid |
| **20** | 10.53-850.5605 | 2.55 | C_46_H_80_NO_8_P | PC (38:6) | 67.98 | 19.48 | 25.37 | phospholipid |
| **21** | 10.99-778.5607 | 1.42 | C_40_H_80_NO_8_P | PC (32:0) | 25.132 | 9.11 | 11.41 | phospholipid |
| **22** | 11.15-878.5920 | 2.03 | C_48_H_84_NO_8_P | PC (40:6) | 42.88379 | 9.37 | 14.01 | phospholipid |
| **23** | 11.27-854.5915 | 2.64 | C_46_H_84_NO_8_P | PC (38:4) | 93.66 | 36.19 | 50.82 | phospholipid |
| **24** | 10.03-745.5497 | 1.48 | C_39_H_77_N_2_O_6_P | SM (34:2) | 23.19 | 6.73 | 8.55 | Sphingolipids |
| **25** | 10.37-747.5655 | 3.08 | C_39_H_79_N_2_O_6_P | SM (34:1) | 93.83 | 23.78 | 41.91 | Sphingolipids |
| **26** | 12.05-855.6601 | 2.24 | C_47_H_91_N_2_O_6_P | SM (42:3) | 50.15 | 9.96 | 12.68 | Sphingolipids |
| **27** | 12.75-831.6597 | 1.67 | C_45_H_91_N_2_O_6_P | SM (40:1) | 29.63 | 7.72 | 8.368 | Sphingolipids |
